# Acne in late adolescence and risk of prostate cancer

**DOI:** 10.1002/ijc.31192

**Published:** 2017-12-14

**Authors:** Henrik Ugge, Ruzan Udumyan, Jessica Carlsson, Ove Andrén, Scott Montgomery, Sabina Davidsson, Katja Fall

**Affiliations:** ^1^ Department of Urology, Faculty of Medicine and Health Örebro University Örebro Sweden; ^2^ Clinical Epidemiology and Biostatistics, School of Medical Sciences Örebro University Örebro Sweden; ^3^ Clinical Epidemiology Unit, Karolinska University Hospital, Karolinska Institutet Stockholm Sweden; ^4^ Department of Epidemiology and Public Health University College London London United Kingdom; ^5^ Department of Medical Epidemiology Karolinska Institutet Stockholm Sweden

**Keywords:** *Propionibacterium acnes*, prostate cancer, acne vulgaris, inflammation, acne vulgaris

## Abstract

Accumulating evidence suggest that *Propionibacterium acnes* may play a role in prostate carcinogenesis, but data are so far limited and inconclusive. The aim of this population‐based cohort study was therefore to test whether presence of acne vulgaris during late adolescence is associated with an increased risk of prostate cancer later in life. We identified a large cohort of young men born in Sweden between 1952 and 1956, who underwent mandatory assessment for military conscription around the age of 18 (*n* = 243,187). Test information along with health data including medical diagnoses at time of conscription was available through the Swedish Military Conscription Register and the National Patient Register. The cohort was followed through linkages to the Swedish Cancer Register to identify the occurrence of prostate cancer until December 31, 2009. We used Cox regression to calculate adjusted hazard ratios (HR) and 95% confidence intervals (95% CI) for the association between acne in adolescence and prostate cancer risk. A total of 1,633 men were diagnosed with prostate cancer during a median follow‐up of 36.7 years. A diagnosis of acne was associated with a statistically significant increased risk for prostate cancer (adjusted HR: 1.43 95%; CI: 1.06–1.92), particularly for advanced stage disease (HR: 2.37 95%; CI 1.19–4.73). A diagnosis of acne classified as severe conferred a sixfold increased risk of prostate cancer (HR: 5.70 95% CI 1.42–22.85). Data from this large prospective population‐based cohort add new evidence supporting a role of *P. acnes* infection in prostate cancer.

## Abstract 2

**What's new?**

Some evidence has suggested that acne vulgaris may increase the risk of prostate cancer, but the data have been inconsistent. The results of this prospective cohort study support a link between acne in adolescence and prostate cancer later in life, potentially implicating the bacteria *Propionibacterium acnes* and resulting inflammation in prostate carcinogenesis. These results also suggest that the association may be stronger for the most severe type of acne and for advanced prostate cancer.

**Abbreviations**
BMI: body mass index
CI: confidence interval
ESR: erythrocyte sedimentation rate
HR: hazard ratio
*P. acnes*: *Propionibacterium acnes*
PIA: proliferative inflammatory atrophy
PIN: prostate intraepithelial neoplasia
PH: proportional hazards

While prostate cancer is the most common cancer among men in the western world, its etiology is still poorly understood. Chronic inflammation is thought to play an important role in the pathogenesis of different types of cancer,^1^ and accumulating evidence suggests a role of inflammation also in prostate cancer.^2^ Both acute and chronic inflammation is often observed in prostate tumor specimens and inflammatory cells are frequently found near areas of proliferative glandular prostate epithelium with the morphological appearance of atrophy.^3^ This type of atrophy (PIA) is thought of as a precursor lesion that may progress to prostate cancer directly or via prostate intraepithelial neoplasia (PIN).^4^

*Propionibacterium acnes,* most commonly associated with acne vulgaris, have been identified as the most prevalent microorganisms in prostate specimens,^5^, ^6^, ^7^, ^8^ and have been associated with presence of inflammatory foci.^9^ We have recently observed that *P. acnes* are more common in prostatic tissue from prostate cancer patients than in samples obtained from men without the disease.^7^ Further, a longitudinal cohort study has demonstrated that men who had received antibiotics as treatment for severe acne were at increased risk of prostate cancer later in life.^10^ While a few other epidemiological studies support a link between acne and prostate cancer,^11^ the literature is inconsistent,^12^, ^13^, ^14^ possibly due to the use of study designs relying on self‐reported exposure information or retrospective data collection. This is to our knowledge the first prospective large‐scale population‐based study using clinically diagnosed acne in order to test if the condition is associated with increased prostate cancer risk.

## Methods

This study is based on a cohort of 284,198 men born between January 1, 1952 and December 31, 1956 that were included in the Swedish Military Conscription Register. These men underwent conscription examinations in the 1970s, except for 80 who were conscripted in 1969 and 1,163 in the 1980s. Examinations were performed at ages 18 and 19 years, with a small number at later ages. At the time, assessment for military service was compulsory in Sweden. Fewer than 4% of the male population were excluded from the enlistment examination due to chronic illness or disability.

**Figure 1:**
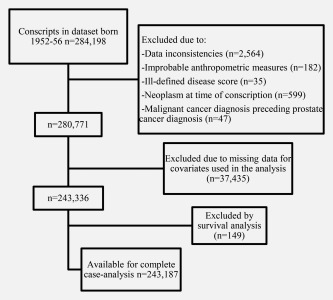
Inclusion and exclusion flow chart.

In the cohort, 2,564 were excluded due to data inconsistencies such as errors in the personal identification number, female sex or uncertain vital status. Further, 182 men were excluded due to improbable measures at the conscription assessment. We also excluded individuals with ill‐defined summary disease score (a standardized score based on presence and severity of health problems, *n* = 35), neoplasms at the time of conscription examination (*n* = 599), and those who had a malignant cancer diagnosis preceding prostate cancer diagnosis (*n* = 47). Next, we excluded men with missing data for covariates used in the analysis (*n* = 37,435), leaving a sample of 243,187 available for complete case analysis (Figure 1).

### Ethical approval

The study was approved by the regional Ethical Review Board, Uppsala, Sweden (decision reference 2014/324).

### Exposure assessment

At the time of conscription examination, participants were subject to a health questionnaire and an interview performed by a medical doctor, who recorded medical conditions according to the 8th edition of the International Classification of Diseases (ICD‐8). Diagnoses were recorded on a practical clinical basis, with emphasis on health problems possibly affecting the participant's performance during military service. Acne was identified using ICD‐8 codes 706.00, 706.01, 706.10, 706.11, 706.12, and 706.20 from the Swedish Military Conscription Register (*n* = 4,847 in the estimation sample) and using ICD‐10 codes L70 from the Swedish National Outpatient register^15^ (added *n* = 189 to the estimation sample). Severe acne was defined using ICD‐8 codes 706.00, 706.01, 706.11, 706.20 and ICD‐10 codes L700C, L70.0 C, L70.1, and L70.2 (see Online Appendix).

### Outcome assessment

Prostate cancer cases were identified using ICD‐7 code 177 from the Swedish National Cancer Register, with a completeness exceeding 95% for common cancer types.^16^ Patients with stages T3–4, N1 or M1 were classified as advanced prostate cancer and stages T1‐T2 as localized prostate cancer. Prostate cancer diagnoses registered before December 31, 2009 (end of follow‐up) were included.

### Other measures

#### Swedish Military Conscription Register

The Swedish Military Conscription Register^17^ records extensive and standardized physical and psychological examinations by physicians and psychologists, as described in detail previously.^18^ Measures included height (cm) and body mass index (BMI) (kg/m^2^), a physical working capacity score (0–9), a cognitive function score (1–9), a stress resilience score (1–9) and a summary disease score (0–9). The physical working capacity score was based on a standardized ergometer test, where the participant was subject to a 5 min sub maximal test, followed by a 5 min maximal test with gradually increasing load. A semistructured interview with a psychologist, covering issues such as emotional stability and social maturity, was used as an assessment of the conscript's potential ability to cope with stress during military service and summarized in a stress resilience score. Cognitive function was obtained from standardized tests covering logical, verbal, technical and spatial abilities. The summary disease score was used to quantify the presence and severity of disease relevant to the capacity to undertake military duty. Erythrocyte sedimentation rate (ESR) was used as a marker of systemic inflammation, and was in the analyses adjusted for erythrocyte volume fraction (EVF).

#### Socioeconomic and demographic characteristics

Demographic data, including information on vital status and emigration, were provided by the government organization Statistics Sweden, using the Total Population Register.^19^ Markers of material and social circumstances in childhood were taken from the 1960 census. Household crowding during childhood was calculated (people/habitable room) and dichotomized. Occupation of the head of household in 1960 was classified as manual, agricultural, farm owners/managers, office workers, business owners/managers and others. Region of residence was taken from the 1970 census.

### Statistical analysis

Descriptive statistics included frequencies, proportions, means and standard deviations. We used time‐dependent Cox‐regression to examine unadjusted and adjusted associations between acne and prostate cancer onset later in life. The functional form of the measures was explored using multivariable fractional polynomial modeling^20^ which indicated a linear relationship with the log hazard of the outcome for height, stress resilience score, cognitive function score, physical working capacity score and erythrocyte volume fraction. The adjusted model included the following measures: birth year, household crowding, head of household's occupation, region of residence, BMI, summary disease score, erythrocyte sedimentation rate (modelled as categorical variables), height, stress resilience score, physical working capacity score, cognitive function score and erythrocyte volume fraction (modeled as linear measures). In sensitivity analysis, we used Cox‐regression to examine the associations with acne identified from conscription records only. The assumption of proportional hazards (PH) for Cox regression was evaluated by using a Grambsch–Therneau test.^21^

The statistical software used was Stata version 14/SE for Windows (StataCorp, College Station, Texas). Tests were two‐sided and statistical significance was defined as ***p*** < 0.05 and 95% confidence intervals that do not include 1.00.

## Results

During follow‐up, 1,633 men were diagnosed with prostate cancer of which 1,317 had localized disease, 203 advanced prostate cancer, and 113 unknown stage. Median follow‐up time was 36.7 years, median age at study‐exit was 55 years and median age at prostate cancer diagnosis was 53 years. Of the 243,187 men eligible for the study, 4,847 (2%) were registered with a diagnosis of acne at time of conscription assessment, likely representing more severe cases. Another 189 men were later diagnosed with acne in the National Patient Register. A slightly higher proportion of men with acne were sons of office workers and a somewhat lower proportion to agricultural workers or farm owners. Men with acne tended to have higher cognitive function and ESR, but lower physical working capacity compared with men without acne. Men with acne further tended to be taller (Table 1). There was no difference in the prevalence of significant health problems or stress resilience in men with and without acne or in stress resilience score (data not shown).

**Table 1:** Characteristics of the exposed and unexposed to acne among Swedish men born 1952–1956 (*n* = 243,187)

Characteristics	No acne *n* = 238,151		Acne *n* = 5,036		*p* a
	*n*	(%)	*N*	(%)	
Head of household's occupation					<0.001
Manual worker	98,525	(41.4)	2,130	(42.3)	
Agricultural worker	9,242	(3.9)	165	(3.3)	
Farm owner/manager	23,696	(9.9)	429	(8.5)	
Office worker	66,320	(27.8)	1,497	(29.7)	
Business owner/managers	25,734	(10.8)	541	(10.7)	
Other/unknown	14,634	(6.1)	274	(5.4)	
Household crowding (persons/room) 1960 census					0.419
>2 people/room	52016	(21.8)	1,076	(21.4)	
BMI categories (kg/m^2^)					0.032
Underweight (<18.5)	27,680	(11.6)	562	(11.2)	
Normal weight (18.5–25.0)	192,519	(80.8)	4,138	(82.2)	
Overweight and obese (>25.0)	17,952	(7.5)	336	(6.7)	
Height (cm)					<0.001
Mean (SD)	178.68 (6.43)		179.57 (6.50)		
Cognitive function score					<0.001
Mean (SD)	5.20 (1.97)		5.38 (1.98)		
Physical working capacity score					0.002
Mean (SD)	6.31 (1.81)		6.23 (1.82)		
Erythrocyte sedimentation rate (mm/h)					<0.001
Median (min–max)	2.00 (1.00–89.00)		3.00 (1.00–48.00)		

a *p* values from *χ* ^2^ test (categorical), *t* test (continuous) or median test (ESR).

We found that men with acne had a statistically significant increased risk of prostate cancer later in life compared with men without acne (HR = 1.43; 95% CI: 1.06–1.92) (Table 2). We further noted a higher magnitude association for men diagnosed with severe acne (adjusted HR: 5.70; 95% CI: 1.42–22.85). We found a modest but statistically significant trend of higher risk with increasing levels of physical capacity. There was no clear association of ESR levels or BMI with prostate cancer risk. When prostate cancer cases were categorized according to disease stage, we found a statistically significant association between acne and advanced prostate cancer (adjusted HR: 2.37; 95% CI: 1.19–4.73). The association with localized disease was of lower magnitude and borderline statistically significant (adjusted HR: 1.39; 95% CI: 1.00–1.94) (Table 3). In a sensitivity analysis, where we excluded men who were diagnosed after conscription (*n* = 189), the overall association was unchanged (adjusted HR: 1.40; 95% CI: 1.06–1.92).

**Table 2:** Hazard ratio (HR) with 95% confidence interval (CI) for the association between acne, and characteristics in late adolescence and prostate cancer among Swedish men assessed for military conscription and born 1952–1956 (*n* = 243,187)

Characteristic	*N* events	Unadjusted HR [95% CI]	Adjusted HR [95% CI]^a^
Acne in and after adolescence			
No acne	1,586	1.00	1.00
Acne	47	1.41 [1.06,1.89]	1.43 [1.06,1.92]
Nonsevere	45	1.37 [1.02,1.84]	1.38 [1.02,1.87]
Severe	2	5.88 [1.47,23.55]	5.70 [1.42,22.85]
Erythrocyte sedimentation rate (mm/h)			
1	439	1.00 [1,00, 1.00]	1.00 [1,00, 1.00]
2–6	1,044	0.90 [0.81, 1.01]	0.93 [0.82, 1.05]
7–10	92	0.79 [0.63, 0.99]	0.83 [0.66, 1.05]
11–14	35	0.95 [0.67, 1.33]	1.02 [0.71, 1.45]
15–89	23	0.73 [0.48, 1.11]	0.80 [0.52, 1.22]
Physical working capacity score (per one unit increase)	‐	1.04 [1.01, 1.07]	1.03 [1.00, 1.06]
BMI (kg/m^2^)			
Underweight (<18.5)	178	0.87 [0.74, 1.02]	0.96 [0.81, 1.13]
Normal weight (18.5–25.0)	1,355	1.00 [1.00, 1.00]	1.00 [1.00, 1.00]
Overweight and obese (>25.0)	100	0.82 [0.67, 1.01]	0.85 [0.69, 1.04]
Household crowding (persons per room)			
<2	1,349	1.00 [1.00, 1.00]	1.00 [1.00, 1.00]
≥2	284	0.77 [0.67, 0.87]	0.86 [0.75, 0.98]

a Adjusting for birth year, head of household's occupation in 1960, household crowding in 1960, height, BMI, physical capacity score, summary disease score, summary cognitive score, stress resilience score, erythrocyte sedimentation rate, erythrocyte volume fraction and region of residence in 1970. The models assume proportional hazards and use 243,187 observations.

**Table 3:** Hazard ratio (HR) with 95% confidence interval (CI) for the association between acne and prostate cancer among Swedish men assessed for military conscription and born 1952–1956 (*n* = 243187), by tumor stage

Exposure	*N* events	Unadjusted HR [95% CI]	Adjusted HR [95% CI]^a^
Advanced prostate cancer			
Acne			
No	194	1.00	1.00
Yes	9	2.21 [1.13,4.32]	2.37 [1.19,4.73]
Localized prostate cancer			
Acne			
No	1,280	1.00	1.00
Yes	37	1.38 [0.99,1.91]	1.39 [1.00,1.94]

a Adjusting for birth year, head of household's occupation in 1960, household crowding in 1960, height, BMI, physical capacity score, summary disease score, summary cognitive score, stress resilience score, erythrocyte sedimentation rate, erythrocyte volume fraction and region of residence in 1970.

## Discussion

In this population‐based study using prospectively recorded data, we observed an association between acne in late adolescence and development of prostate cancer later in life. The data suggest that the association may be stronger for the most severe type of acne and for advanced prostate cancer.

The literature is currently limited and somewhat conflicting regarding the association between acne and prostate cancer. The results from a prospective cohort study suggest that acne during young adulthood is associated with an increased risk of prostate cancer‐specific death.^11^ Another cohort study using prospectively collected data shows an increased prostate cancer risk for men who reported treatment with tetracycline for more than four years as a marker of severe acne.^10^ A few case‐control studies, where subjects have been asked regarding their history of acne, have on the other hand not shown any associations between acne and prostate cancer,^12^, ^13^, ^14^ and one case–control study reported an inverse association between acne‐related facial scarring and later prostate cancer.^22^ In the same cohort, high serum titers of antibodies directed against *P. acnes* were observed to be inversely associated with prostate cancer risk.^23^ Studies using retrospectively collected and self‐reported data are sensitive to exposure misclassification, which may explain the reported null findings.^12^, ^13^, ^14^, ^22^ Reliance on self‐reported information on acne has also been shown to be a less reliable measure of the disease.^24^

The prevalence of acne in this cohort (2%) may seem low compared with other studies, and compared with the overall incidence of acne among adolescent men. Acne vulgaris is considered to affect a majority of, if not all, adolescents to some extent, and some 20% are said to be affected by moderate to severe lesions.^25^, ^26^ The latter figure corresponds roughly to the number counted as exposed in other studies examining the relationship between self‐reported history of acne and prostate cancer.^12^, ^13^, ^14^ It is however likely that men registered with acne by a physician in the setting of conscription examination constitute a group with more severe acne. The prevalence of severe acne based on physical examinations in similar settings has earlier been estimated to 2.9–6.9 percent.^27^, ^28^ While the present study may underestimate the number of exposed, it should accurately have identified clinically significant cases of acne. The other study reporting a positive association between acne and prostate cancer^10^ could be argued to have used a similarily strict definition of exposure since prescription of tetracycline would have required physical examination. Identification of more severe cases may thus help explain discrepancies with results of studies based on self‐reported, less severe acne. Indeed, although based on very small numbers, our results suggest a stronger link with what specifically has been classified as more severe forms of acne. The focus of the study is further on acne later during adolescence (age 18–19 years), and the results may not be generalizable to persons with acne earlier in life.

*P. acnes* can induce inflammatory reactions, through complement activation and induction of proinflammatory cytokines.^29^, ^30^ Induction of cytokines such as IL‐6 and IL‐8, partly through activation of toll‐like receptors (TLRs), is thought to be involved in the pathogenesis of acne,^31^ and has been demonstrated to occur in both skin and prostate cells infected by *P. acne*.^7^, ^32^, ^33^ Although the exact mechanism of *P. acnes*‐associated pathogenesis is not completely understood in either skin or prostate, there is evidence pointing to the importance of the host inflammatory response.^32^, ^34^ *P. acnes* is ubiquitous on the skin, but only a smaller proportion of the population develop severe acne lesions as a consequence of colonization, which further suggests that individual variation in immune response may influence the outcome of infection.^35^ Immunologic phenotypes predisposing an intense dermatologic reaction could also be prone to a similar inflammatory response in the prostate. We found that the mean erythrocyte sedimentation rate (ESR), a marker of systemic inflammation, was higher among men with than without acne, but not an independent determinant of prostate cancer risk.

An alternative explanation of our result may involve androgens. Hormonal activity plays a role in the pathology of acne^36^, ^37^ and the central role of androgen signalling in prostate cancer and prostate cancer treatment has been known for a long time.^38^ The role of androgen signalling in prostate carcinogenesis is however not as clearly established.^39^ We were unfortunately not able to address this potentially important confounder in this study, which is a limitation.

The strengths of this study include its large study population, prospective design and long, essentially complete, follow‐up. Another advantage is that the exposure, the diagnosis of acne, was made as part of the physical examination during the assessment for conscription and collected independently of a later diagnosis of prostate cancer. A weakness is that the number of exposed was low and the power for detailed analyses limited. The study population is furthermore relatively young which results in a low number of prostate cancer cases, possibility leading to an underestimation of the true association. Specifically, our study could be said to have examined the association between acne in late adolescence and *early* prostate cancer diagnosis. Whether the association remains for prostate cancer at later ages is unknown.

In conclusion, the results of this prospective cohort‐study indirectly support the hypothesis that *P. acnes* may play a role in prostate cancer. Whether the association is explained by *P. acnes*, host factors associated with specific immune responses, hormonal or other factors remains to be investigated further.

## Supporting information

Supporting Information
