# Supplementary material for: Acne in late adolescence and risk of prostate cancer
Source: Int J Cancer. 2017 Dec 14;142(8):1580–5. doi: 10.1002/ijc.31192 (PMC5838533; doi:10.1002/ijc.31192)
Supplement: Supplementary file 1 — Supporting Information [file IJC-142-1580-s001.docx]

| **Appendix: list of ICD-codes (8th and 10th version respectively) used to identify acne from conscription register and out-patient register** | | | |
| --- | --- | --- | --- |
|  |  |  |  |
|  |  |  |  |
|  |  |  |  |
| **Variable** | **Code** | **Description** |  |
|  |  |  |  |
| **Acne** |  |  |  |
| **ICD-8:** |  |  |  |
|  | 706.00 | Acne necrotica |  |
|  | 706.01 | Acne varioliformis, unspecified |  |
|  | 706.10 | Acne vulgaris |  |
|  | 706.11 | Acne cheloides. Acne scleroticans nuchae | |
|  | 706.12 | Acne alia sive, unspecified |  |
|  | 706.20 | Cysta sebacea |  |
| **ICD-10:** | |  |  |
|  | L70 | Acne |  |
|  | L70.0 | Acne vulgaris |  |
|  | L70.0A | Acne vulgaris comedonica |  |
|  | L70.0B | Acne vulgaris papulopustulosa |  |
|  | L70.0C | Acne vulgaris nodulocystica |  |
|  | L70.0W | Annan acne vulgaris |  |
|  | L70.0X | Acne vulgaris, unspecified |  |
|  | L70.1 | Acne conglobata |  |
|  | L70.1A | Acne fulminans |  |
|  | L70.1X | Acne conglobata, unspecified |  |
|  | L70.2 | Acne varioliformis |  |
|  |  |  |  |
| **Severe acne** | |  |  |
| **ICD-8:** |  |  |  |
|  | 706.00 | Acne necrotica |  |
|  | 706.01 | Acne varioliformis, unspecified |  |
|  | 706.11 | Acne cheloides. Acne scleroticans nuchae | |
|  | 706.20 | Cysta sebacea |  |
| **ICD-10:** | |  |  |
|  | L70.0C | Acne vulgaris nodulocystica |  |
|  | L70.1 | Acne conglobata |  |
|  | L70.1A | Acne fulminans |  |
|  | L70.1X | Acne conglobata, unspecified |  |
|  | L70.2 | Acne varioliformis |  |
